# Dr. Arnott's Elements of Physics

**Published:** 1830-04-01

**Authors:** 


### Elements of Physics, or Natural Philosophy, &c. By *Neil Arnott*, M.D. Volume the Second, 1830.

We cannot too strongly recommend to our medical readers these two valuable and eloquent volumes, in which many of the most important branches of human knowledge are conveyed in language so elegant, and in terms so easy of comprehension, that he who runs may read, and whoever reads must understand. These volumes cannot fail to generate and diffuse an ardent desire for the study of natural philosophy—the most delightful, as well as the most comprehensive, circle in the sciences. We can only introduce two or three extracts, to exemplify the manner in which Dr. Arnott has contrived to invest physics with the charms of poetry or romance, and to melt down rugged and uncouth technicalities into language the most smooth, energetic, and harmonious.

### 1. Influence of Temperature on Decomposition.

"Again, as regards dead animal substances, we find that although at a certain, not very elevated, temperature, they undergo that change in the relations of their elements which we call putrefaction, when nearly their whole substance rises again to form part of the atmosphere, still at or below the temperature of freezing water, they remain unaltered for any length of time. In the middle of summer, recently caught salmon, or other fish, packed in boxes with ice, is conveyed fresh from the most remote parts of Britain to the capital. In our warmest weather, any meat or game may be long preserved in an ice-house. In Russia, Canada, and other northern countries, on the setting in of the hard frosts, when the inferior animals have difficulty in finding food, the inhabitants kill their winter supply, and store their provender of frozen flesh or fowl, as in other countries men store that which is salted or pickled. But the most striking instance of this kind we can adduce is the fact, that on the shore of Siberia, in 1801, in a vast block or island of ice, of which the surface was then more melted than in preceding summers, the carcase of an antediluvian elephant was found, perfectly preserved—an elephant differing materially from those now existing on earth, but its skeleton exactly corresponding with the specimens found deep buried in various countries. The creature was soon discovered by the hungry bears of the district, which were seen tearing off its hairy hide, and feeding on its flesh, as fresh almost as if it had lived yesterday, although it must have been of an æra long anterior to that of any existing monument on earth, of human art, or even of human being. Long after it fell from the ice to the sandy beach, and when its tusks had been carried away for sale by a Tungusian fisherman, and its flesh had been nearly devoured, a naturalist who visited it found an ear still perfect, and its long mane, and part of its upper lip, and an eye with the pupil yet distinguishable, which had opened on the glories of a former or younger world! About 30 lbs. weight of its hair, which had been trodden into the sand by the bears while eating the carcase, was collected, and is now preserved in different museums of natural curiosities—some, for instance, in the museum of the London College of Surgeons." 120.

The influence of heat on the animal and vegetable world, is treated by our eloquent and highly informed author with interest and animation. We shall make room for one short extract.

### II. Solar Heat on Animate and Inanimate Nature.

"The influence which heat exerts on inanimate nature, is more immediately and completely perceived by the common mind, than its influence on beings which have life. Thus to all it is obvious, that the contrast between a winter and summer landscape, is owing chiefly to the effect of heat on the water of the landscape;—that during its absence in winter, there is the dry barren deformity of accumulated ice and snow, covering every thing, the roads impassable, the rivers bound up, perhaps hidden, the air deprived of moisture, and loaded often with powdery drift;—and that when warmth comes, the living streams again appear, gliding their way, the cascades pour, the rills murmur, the canals once more offer their bosoms to the boats of commerce, the lake and pool again show their level face, reflecting the glories of the heavens, and the genial shower falls upon the bosom of the softened earth, become ready to receive the spade or the ploughshare. Now this change is not at all greater than what happens to a winter tree acted upon by the warmth of spring.—To take another instance from inanimate nature, it may be said with truth, that heat applied to the cold boiler of a steam-engine, is the cause of all its succeeding motions; of the heaving of its beam and pumps, the opening and shutting of its valves, the turning of its wheels, and its ultimate performance of work, as of spinning, or weaving, or grinding, or propelling vehicles by land and water: but as truly may it be said, that heat coming to a seed which has lain cold for ages, is the cause of its immediate germination and growth; or coming to a lately frozen tree is the cause of the rising of its sap, the new budding and unfolding of its leaves and blossoms, the ripening of its fruit. And what is true of one seed or tree, is true of the whole of the vegetable creation. When the warm gales of spring have once breathed on the earth, it soon becomes covered, in field and in forest, with its thick garb of green, and soon opening flowers or blossoms every where breathe back again a fragrance to heaven. Among these the heliotrope is seen always turning its beautiful disc to the sun, and many delicate flowers only open their leaves to catch the direct solar ray, but close them often even when a cloud intervenes, and certainly when the chills of night approach. On the sunny side of a hill, or in the sheltered crevice of a rock, or on a garden wall with warm exposure, there may be produced grapes, peaches, and other delicious fruits, which will not grow in situations of an opposite character—all acknowledging heat as the immediate cause, or indispensable condition, of vegetable life.

"But among animals, too, the effects of heat are equally remarkable. The dread silence of winter, for instance, is succeeded in spring by one general cry of joy. Aloft in the air the lark is every where carolling; and in the woods and shrubberies, a thousand little throats are similarly pouring forth their songs of gladness—during the day, the thrush and blackbird near our dwellings, are heard above the rest, and with the evening comes the sweet nightingale;—for all of which it is the season of love and of exquisite enjoyment. And it is equally so for animal nature generally: in favoured England, for instance, in April and May the whole face of the country resounds with lowings and bleatings and barkings of joy. And even man, the master of the whole, and whose mind embraces all times and places, is far from being insensible to this change of season. His far seeing reason of course draws delight from the anticipation of autumn, with its fruits; and his benevolence rejoices in the happiness observed among all inferior creatures; but independently of these considerations, on his own frame the returning warmth exerts a direct influence. In early life, when the natural sensibilities are yet fresh and unaltered by the habits of artificial society, spring to man is always a season of delight. The eyes brighten, the whole countenance is animated, and the heart feels as if new life were come, and has longings for fresh objects of endearment. Of those who have passed their early years in the country, or, among the charms of nature, as contrasted with the arts of cities, there are few who, in their morning walks in spring, have not experienced without very definite cause, a kind of tumultuous joy, of which the natural expression would have been, how good the God of nature is to us! Spring is a time when sleeping sensibility is roused to feel that there lies in nature more than the grosser sense perceives. The heart is then thrilled with sudden ecstacy, and wakes to aspirations of sweet acknowledgment." 124.

We regret that we have not space for the introduction of a few more specimens of Dr. Arnott's manner and matter. His style is peculiarly well fitted for the diffusion of scientific knowledge among popular readers; and we hope he will not allow his talents, which are of a high order, to lie inactive, now when his moral and physical powers are in their prime.

